# Data from two different culture conditions of *Thalassiosira weissflogii* diatom and from cleaning procedures for obtaining monodisperse nanostructured biosilica

**DOI:** 10.1016/j.dib.2016.05.033

**Published:** 2016-05-28

**Authors:** Danilo Vona, Laura Urbano, Maria A. Bonifacio, Elvira De Giglio, Stefania Cometa, Monica Mattioli-Belmonte, Fabio Palumbo, Roberta Ragni, Stefania R. Cicco, Gianluca M. Farinola

**Affiliations:** a Department of Chemistry, University of Bari “Aldo Moro”, Via E. Orabona, 4, 70126 Bari, Italy; b Jaber Innovation s.r.l., Via Calcutta, 8, 00100 Roma, Italy; c Department of Clinical and Molecular Sciences, Università Politecnica delle Marche, Via Tronto, 10/a, 60020 Ancona, Italy; d CNR NANOTECH, Department of Chemistry, University of Bari “Aldo Moro”, Via E. Orabona, 4, 70126 Bari, Italy; e CNR- ICCOM, Department of Chemistry, University of Bari “Aldo Moro”, Via E. Orabona, 4, 70126 Bari, Italy

**Keywords:** Biosilica, Diatoms, Cell growth, Biomaterials

## Abstract

Diatoms microalgae produce biosilica nanoporous rigid outershells called frustules that exhibit an intricate nanostructured pore pattern. In this paper two specific *Thalassiosira weissflogii* culture conditions and size control procedures during the diatoms growth are described. Data from white field and fluorescence microscopy, evaluation of cell densities and cell parameters (*k* value and *R* value) according to cell culture conditions are listed. Different cleaning procedures for obtaining bare frustules are described. In addition, FTIR and spectrofluorimetric analyses of cleaned biosilica are shown.

The data are related to the research article “*Chemically Modified Diatoms Biosilica for Bone Cell Growth with Combined Drug-Delivery and Antioxidant Properties*” [1].

**Specifications Table**

**Table:** 

Subject area	*Chemistry*, *Biology*, *Phicology*
More specific subject area	*Diatoms*, *Biomaterials*
Type of data	*Images*, *text file*, *graph*, *figure*
How data was acquired	*FT-IR spectra were recorded on a PerkineElmer* 1710 *spectrofotometer using dry KBr pellets*
	*Fluorescence images were recorded on an Axiomat microscope*, *Zeiss* (*German*), *fluorescence filter set* 15 *(exc.*546 nm,*em*.590 nm)
	*Fluorescence spectra were recorded on a Varian Cary Eclipse spectrofluorimeter*
	*Cell densities and cell parameters were taken using Burker hemocytometer Knittel Glass*
Data format	*Analyzed*
Experimental factors	*Diatoms culturing in f*/2 *Guillard-sea water enriched medium and cleaning procedure via different acidic-oxidative treatments*
Experimental features	*Characterization of biosilica via FT*-*IR and spectrofluorimetry*
Data source location	*Dipartimento di Chimica, Università degli Studi* “*Aldo Moro*”, *Bari*, *Italia*
Data accessibility	*Data are provided with this article*

**Value of the data**

• Easily achievable conditions for growing diatoms cells and cleaning procedures for the biosilica extraction from the living cultures will be helpful for researchers without specific biological background.
• Evaluation of cell densities and cell parameters according to the two living cell cultures conditions are reported for a basic biological monitoring.
• These datasets are useful for obtaining monodisperse biosilica in high yields and could be helpful for the developing science of bionanotechnology.

## 1 Data

**Fig. 1:**
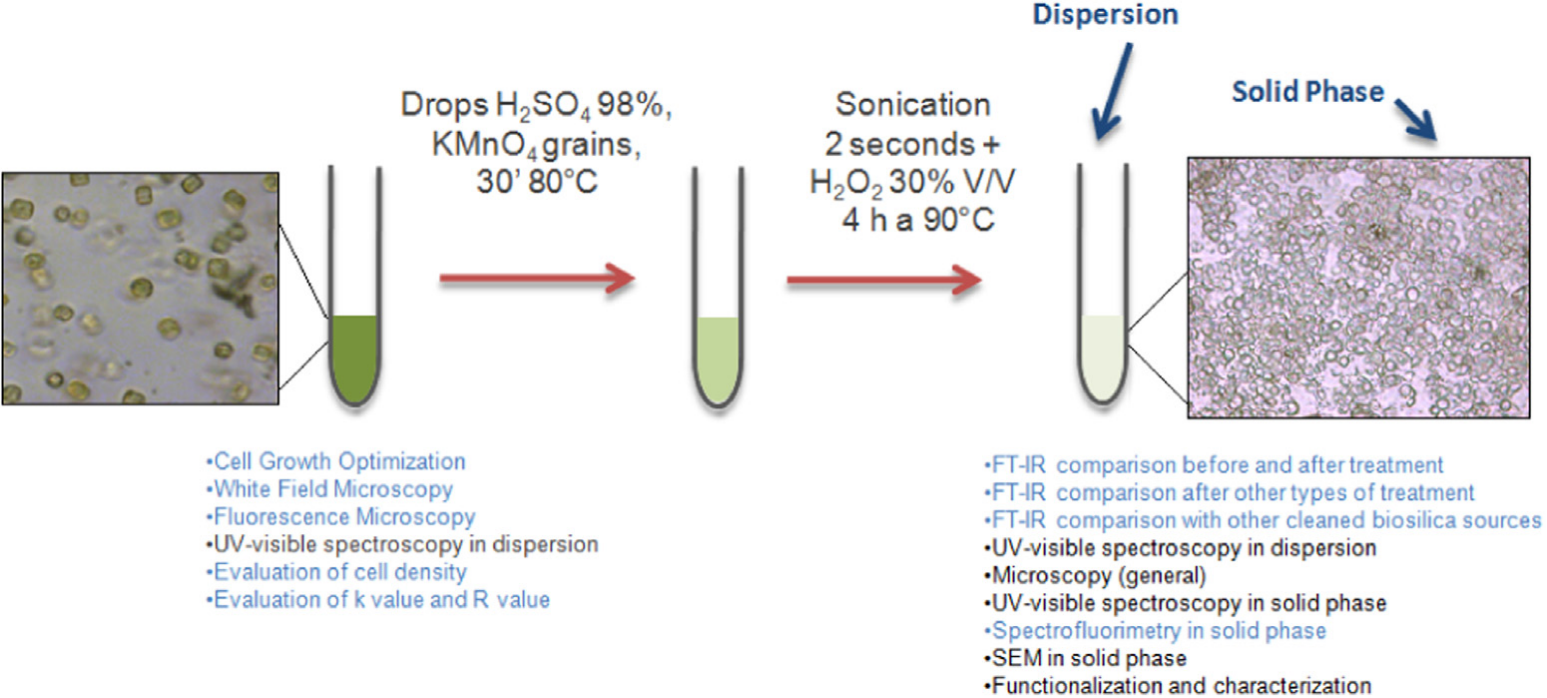
From the living T. weissflogii to solid pure biosilica. This general scheme summarizes all the steps useful to obtain cleaned biosilica from living *T*. *weissflogii* diatoms.

**Fig. 2:**
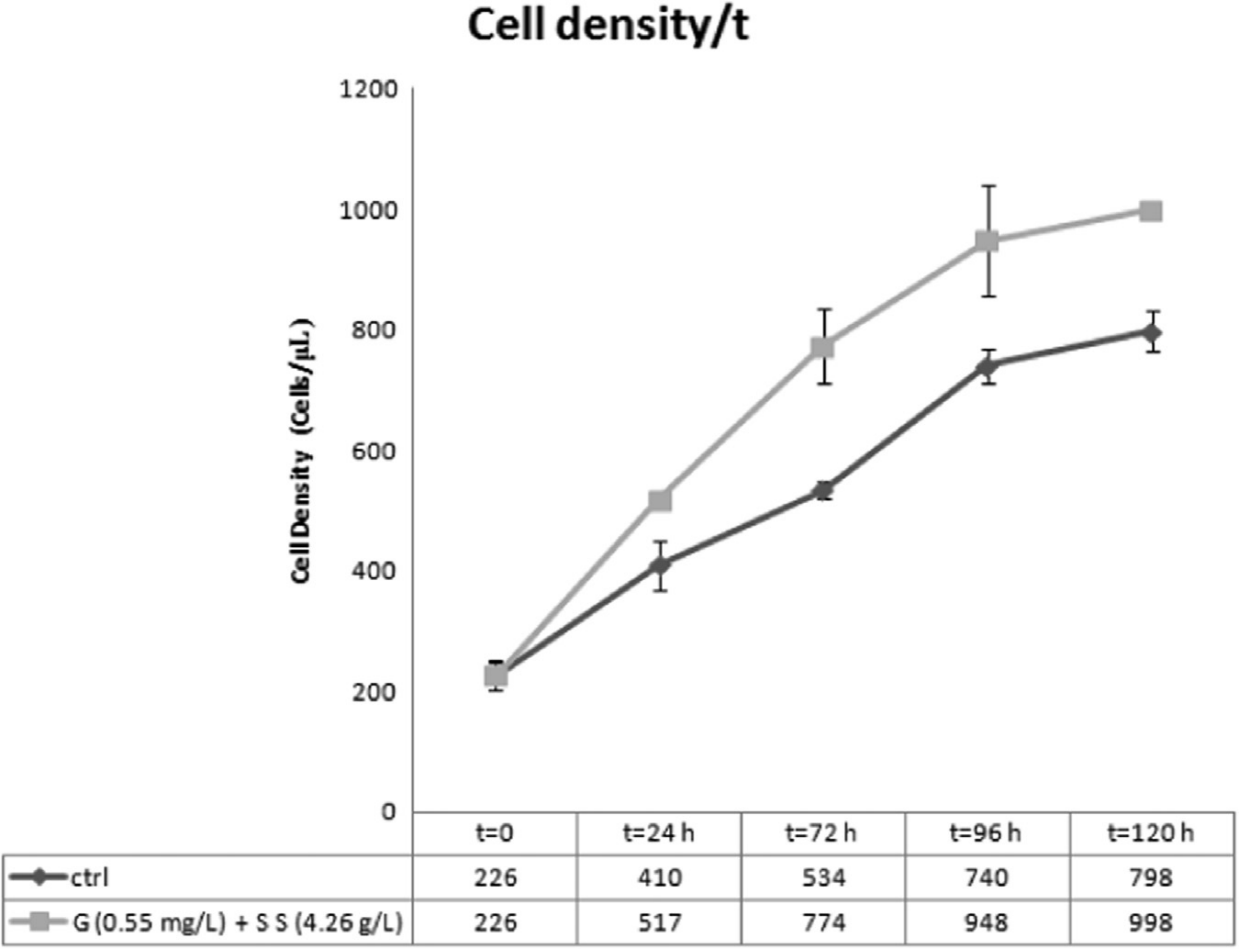
Cell density evaluations for cell parameters (*k* value and *R* value) according to the growth of diatoms without ([ctrl] sample) and with (G+SS) a mixture of glucose (0.55 mg L^−1^) and sodium sulfate (4.26 gL^−1^), in a timing of 120 h.

**Fig. 3:**
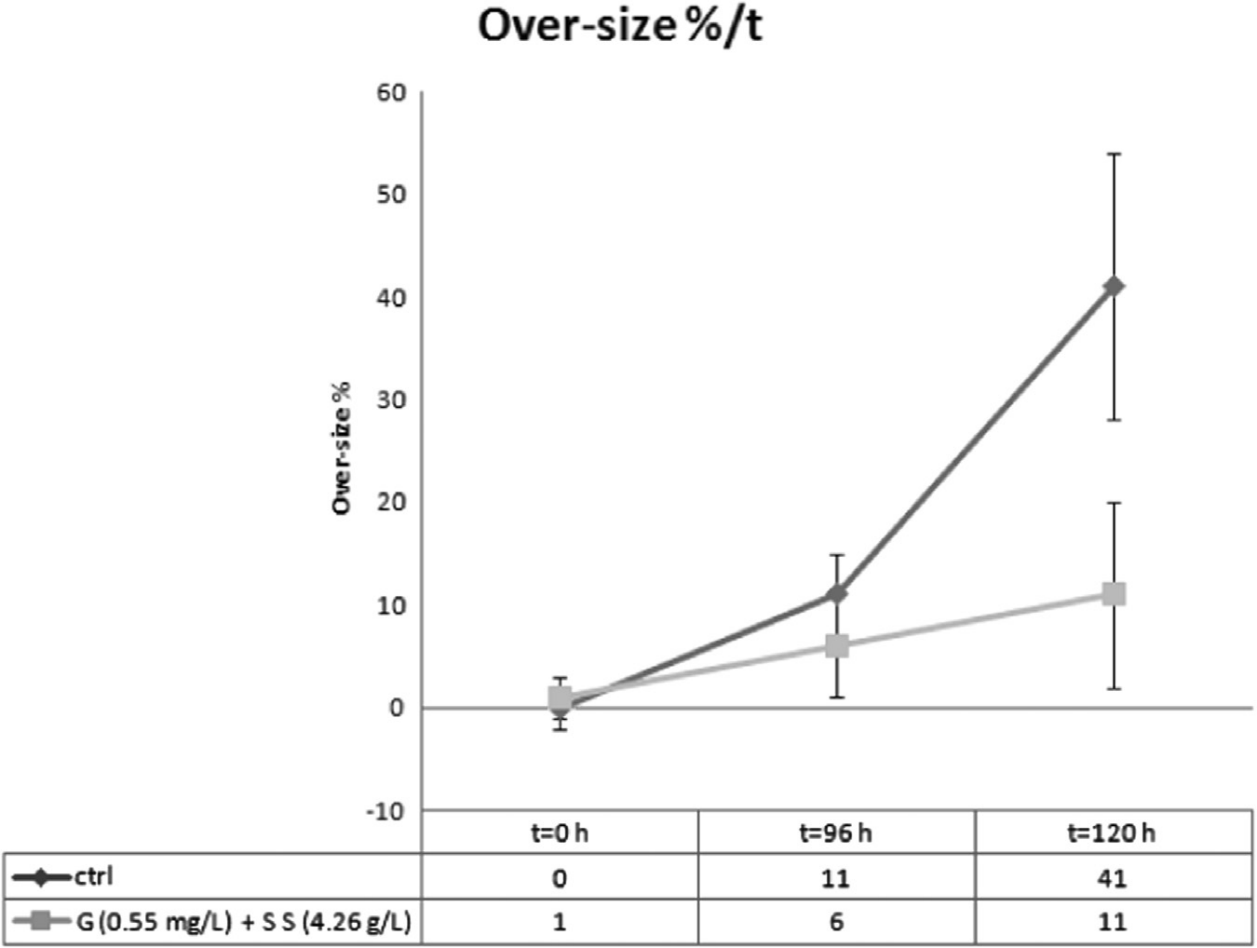
Over-size percentage (%) in 3 times (0, 96 and 120 h) for both [ctrl] and [G+SS] cultures. The over-size percentage is the ratio between number of cells with valve diameter >11 μm and the number of total cells.

**Fig. 4:**
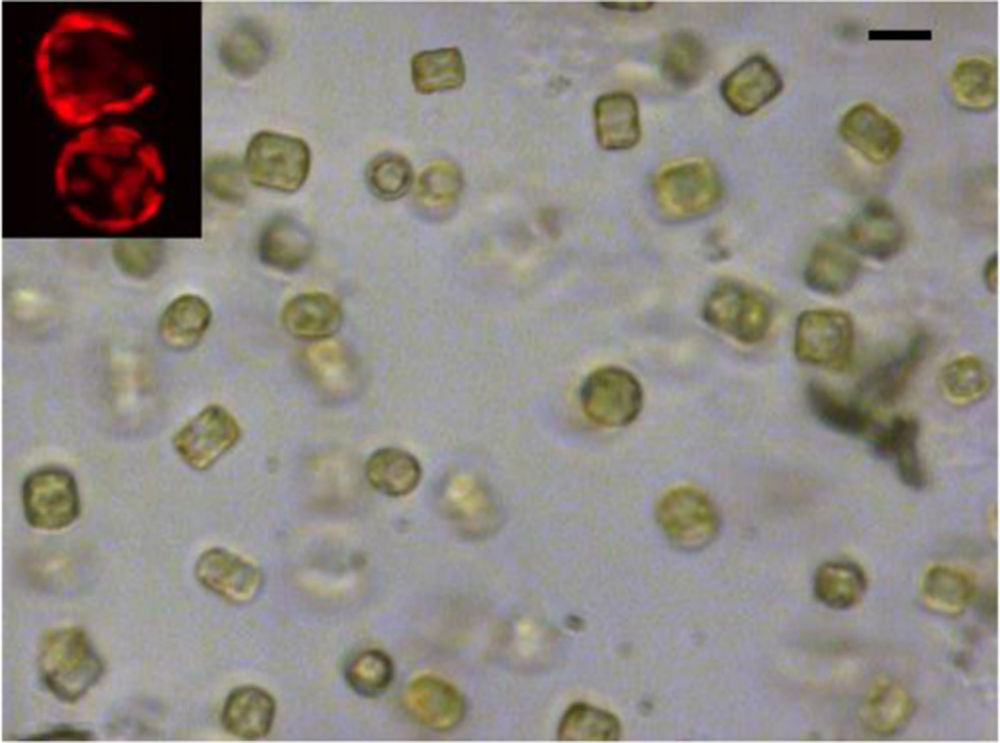
White Field Microscopy Images (in transmission, 40x) of living *T*. *weissflogii* cells in culture; Bidimensional Fluorescent Microscopy (in reflection, 60x, inset) of living *T*. *weissflogii* cells with red emissive organized chloroplasts. Marker: for the main picture, 10 μm; for the inset, 5 μm.

Data provided in this article represent the results from two different cultures conditions of *Thalassiosira weissflogii* diatoms. In Fig. 1, all the steps to obtain cleaned biosilica from living cells are summarized. Figs. 2, 3 and 4 present cell density evaluations in the living cultures and related cell size control according to the different cultures conditions.

**Fig. 5:**
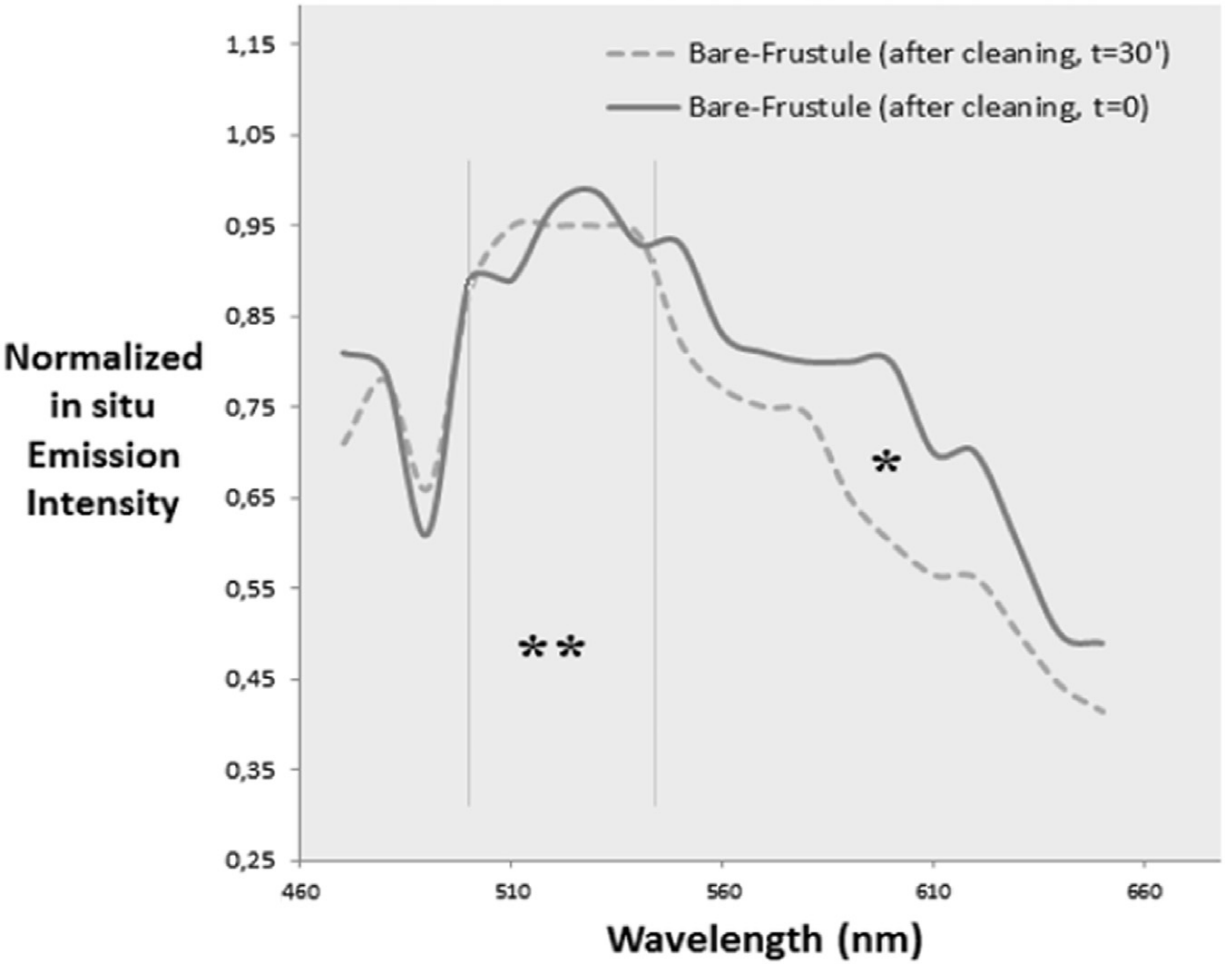
Time-lapse photoluminescence study (*λ* excitation 385 nm).

FTIR signals of extracted biosilica are also shown (Table 1) together with the emission spectra of the bare frustules (Fig. 5).

## 2 Experimental design, materials and methods

We analyzed each step of the *T*. *weissflogii* growth by white field and fluorescence microscopy and we evaluated cell density and cell parameters for cell viability estimation. The living diatoms underwent different acidic/oxidative cleaning procedures and FTIR analyses and UV–vis spectra were done on the solid biosilica obtained.

### 2.1 Diatoms cultures conditions

The diatom *T*. *weissflogii* (culture collection of algae and protozoa, CCAP strain 1085/10) was grown in a sterile f/2 Guillard enriched by seawater medium in PS flasks (25 mL) [1]. A preparation of stock solutions was performed adding 0.2 g/100 mL of NaCl in sea water (3.8–3.875% of salinity rate), and bufferring with NaOH (2 N) till pH value of 8 [2] The medium was enriched with Na_2_SiO_3_·9H_2_O, trace metals, and a vitamin mix. [3] The cultures were aerated manually 2 times per day, to provide air and to prevent algae precipitation. In the first 4 days of subculture glucose was added (0.55 mg L^−1^) for enhancing cell viability, and sodium sulfate (4.26 gL^−1^) for increasing photosynthesis yields, as reported in the literature. [4] Moreover, in order to avoid bacterial contamination, a low amount of kanamycin (0.5 mg L^−1^) was added. Growth was controlled at 18–20 °C under a continuous photon flux density (PFD) provided by cool-white fluorescent tubes. The light source was placed 15 cm away from cultures. The light/dark cycle was 12 h illumination/12 h darkness and minimal air change (basal oxygen influx) was guaranteed by ventilation through sterile filters applied onto tubes.

### 2.2 Microscopy of living *T*. *weissflogii* diatoms

A 10 μL droplet of culture was spotted on the cover slip and a glass slide was placed on. After removing the surplus medium with cotton, the enamel seal was put between the cover slip and the glass slide. Living *T*. *weissflogii* diatoms appeared as box-like structures in which the green chloroplasts mottles were evident and compartmentalized close to the glass-box frustule (white field transmission microscopy, Fig. 4). Green mottles were red fluorescent when observed using a bidimensional fluorescent microscope (in reflection, inset from Fig. 4). The control of the cell density was closely related to the fluorescence and white field microscopies: vital cells appeared green (evident yellowish-white mottles were instead evident in chlorotic state), with intact box-like structures and with high qualitative levels of red emission from chloroplasts.

### 2.3 Evaluation of cell densities and cell parameters according to cell culture conditions

We evaluated cell density using standard counting in a Burker hemocytometer (by monitoring the first 5 days, when cell density reached $5-8{x10}^{5}$ cells mL^−1^) taking into account that cells normally were sub-cultured after 14–15 days of growth. In the specific way, we analyzed cell density and cell parameters (*k* value and *R* value) according to the growth of diatoms without (control sample [ctrl]) and with the addition of a energizing mixture containing glucose (0.55 mg L^−1^) and sodium sulfate (4.26 gL^−1^) [G+SS]. So we performed cell density experiments for [ctrl] sample and [G+SS] sample, and their results are reported in Fig. 2. We calculated *k* values (number of generations) for both the samples, admitting a general binary scission in *T*. *weissflogii* growth. The *k* value was obtained from this formula:

$${k=\text{logN}}_{t}-{\mathrm{logNt}}_{o}/0.301$$

We analyzed cultures with a Nt_o_ (starting cell densities) of 226 cells/μL, in a total time of 120 h. So we calculated a *k*′ value for the [ctrl] and a *k*″ value for the [G+SS] culture, considering *k*′ and *k*″ as number of generations in the time *t* (and considering the respective Nt values as final cell densities, as reported in Fig. 2):

$$k^{\prime }=\;\log\left(798\;\text{cells}/\mu L\right)-\;\log\left(226\;\text{cells}/\mu L\right)/0.301=2.90-2.35/0.301=1.82k^{\prime \prime }=\;\log\left(\text{998}\;\text{cells}/\mu L\right)-\;\log\left(226\;\text{cells}/\mu L\right)/0.301=2.99-2.35/0.301=2.13$$

We calculated also the *R* values (growth velocity of the cultures), obtaining *R*′ value for the [ctrl] and *R*″ value for the [G+SS] culture, in which:

$$R=k/t=0\text{.015generations}/hR^{\prime }=k^{\prime }/t=0\text{.018generations}/h$$

These cells parameters confirmed that glucose and sodium sulfate are considered as energizing nutrients which enhance cell growth (*R*′>*R*).

Glucose and sodium sulfate were also considered controllers for the over-sizing (which is the unbalanced growth in size). We monitored the over-size percentage (%) in 3 times (0, 96 and 120 h, Fig. 3), which is the ratio between number of cells with valve diameter >11 μm and total cells, for both [ctrl] and [G+SS] cultures. Glucose and sodium sulfate allowed us to obtain a quasi mono-dispersed diatoms in cultures. Generally [G+SS] cultures do not exhibit cells with valve diameter <10 μm.

### 2.4 Cleaning procedures of diatoms cultures

a. Cleaning with trifluoroacetic acid (TFA-acid): a 5 mL suspension of cells was collected by centrifugation (900 rpm×15′). After the removal of the supernatant 100 μL of H_2_O Millipore were added to rinse pellet; the procedure started adding 3 drops of trifluoroacetic acid (TFA) and 20 μL KMnO_4_+20 μL H_2_O_2_ (very low amount of oxidant only to spark cleaning reaction), and the pellet was kept at 90 °C for 5 h; the procedure continued with sonication for 5″, and cleaned diatoms are pelletted at 1000 rpm for 30′. A series of washing steps was then performed (3x H_2_O Millipore), and pellet was suspended in 500 μL of pure EtOH.
b. Cleaning with hydrochloric acid and methanol (HCl+meth): according to the literature [5], removal of organic matter was performed by several washes with 50/50 HCl/deionised water, deionised water and methanol. A pellet of diatoms coming from a 5 mL suspension in culture was suspended in 50:50 HCl/deionised water for 1 min and then centrifuged at 1100 rpm for 5 min. Pellets were suspended in 50/50 HCl/deionised water for 1 min and centrifuged at 1150 rpm for 6 min, then again in 50/50 HCl/deionised water for 1 min and centrifuged at 1150 rpm for 12 min. This cycle of HCl washes was followed by three steps in deionised water. Lastly, samples were suspended in methanol for 2 min and centrifuged at 800 rpm for 10 min. The resulting pellet was dried using a pump and appeared as white. Samples were weighted, sealed and stored at 4 °C. This cleaning procedure was reported to be efficient in a soft organic matter removal from entire frustules, avoiding the entropic opening in valves and girdle.
c. Cleaning with hydrochloric acid-hydrogen peroxide and methanol (HCl+meth+H_2_O_2_): as the previous method, with the difference that all the washing steps were performed with HCl:H_2_O_2_ deionized water (15:30:55).
d. Cleaning with sulphuric acid, potassium permanganate and hydrogen peroxide (H_2_SO_4_+KMnO_4_+H_2_O_2_): cells were previously collected by centrifugation (1000 rpm, 20′) from a 5 mL suspension of living cultures, rinsed with bidistilled water (total volume of 200 μL) and organic matter was removed through a mix of acid treatment and oxidation with H_2_SO_4_ (5 drops, 98% w/w, 1 drop of HCl 37% w/w) and KMnO_4_ (2 grains from solid powder) at 80 °C for 30′; after 2 s of sonication, a further oxidation step with hydrogen peroxide (200 μL, 30% w/w) at 90 °C for 4 h was performed. [6] This treatment was followed by repeated washing steps with bidistilled water and soft centrifugations (1100 rpm, 10′). This cleaning procedure was reported to be the most efficient in a hard organic matter removal from entire frustules, even if the entropic opening of frustules in valves and girdle occurred.

### 2.5 Biosilica deposition on glass slides

After the cleaning procedure, we deposed biosilica dispersion on glass. The glass was pre-treated with a H_2_SO_4_ (1 mL, 98% w/w)-hydrogen peroxide (2 mL, 30% w/w) at 80 °C for 1 h. After this pre-treating, the glass slide was dried. A 20 μL whitish dispersion in water was put with 20 μL of acetone in eppendorf. Then 15 µL of the bottom enriched part of whitish pellet from the eppendorf were put on a cleaned glass slide. A pre-annealing at 60 °C for 10–30′ was useful to dry the sample. If necessary (for multilayer sample), this deposition was repeated together with the pre-annealing. A microscopy monitoring was necessary to check layer density and layer quality. If the deposed frustules did not appear transparent, a further washing of the pellet with a solution 1:1 acetone and DMSO was sufficient for a successful new deposition. A final thermal treatment at 120-200 °C for 2 h on heating plate was performed to make samples dry and to link silica shells onto glass slide surface. For the dried frustules sample preparation, after soft ethanol rinsing (50 µL on the white spots) and drying, diatoms shells remained intact and attached onto glass slide surface, and they were ready for further investigations.

### 2.6 Time-lapse photoluminescence analyses (*λ* excitation 385 nm)

Using an UV-excitation wavelength (385 nm), we recorded emission spectra of cleaned dried frustules (layered on glass slides) before first exposition (*t*=0) and 30′ after first exposition (*t*=30′). Results showed that there was a general quenching of fluorescence after 550 nm (*, Fig. 5), while the blue-green area of the spectra remained stable (**, Fig. 5). This quenching occurred after 30′ (first UV exposition) and it was maybe due to a photo-degradation of such fluorophores [7]. The blue-green area (**, Fig. 5) remained stable and not quenched [8].

## Figures and Tables

**Table 1:** FTIR signals of biosilica obtained by cleaning procedure (d).

**ν (cm-1)**	**Functional group**	**Type of signal**
3402	OH (Water)	stretching
2927–2921	CH	stretching asymm. symm.
1640	OH (Silanole)	stretching
1175	Si-O (Si–O–Si)	stretching asymm.
1066–1009	Si-O (Si–O–C)	stretching
884	Si-O (Si–O–Si)	stretching symm.
584–450	Si-O (Si–O–H)	stretching

## References

[1] Cicco S.R., Vona D., De Giglio E., Cometa S., Mattioli-Belmonte M., Palumbo F., Ragni R., Farinola G.M. (2015). Chemically modified diatoms biosilica for bone cells growth with combined drug delivery and antioxidant properties. ChemPlusChem.

[2] Guillard R.R.L., Ryther J.H. (1962). Studies of marine planktonic diatoms: *I. Cyclotella* nana hustedt, and *Detonula confervacea* (cleve) gran. Can. J. Microbiol..

[3] Coombs J., Darley W.M., Holm-Hansen O., Volcani B.E. (1967). Studies on the biochemistry and fine structure of silica shell formation in diatoms. Chemical composition of *Navicula pelliculosa* during silicon-starvation synchrony. Plant Physiol..

[4] Radchenko J.G., Il’yash L.V., Fedorov V.D. (2004). Effect of exogenous glucose on photosynthesis in the diatom *T*. *weissflogii* depending on nitrate nitrogen supply and illumination. Biol. Bull. Russ. Acad. Sci..

[5] Lang Y., del Monte F., Rodriguez B.J., Dockery P., Finn D.P., Pandit A. (2013). Integration of TiO_2_ into the diatom *T*. *weissflogii* during frustule synthesis. Sci. Rep..

[6] Throndsen J. (1978). Preservation and storage. Phytoplankton Manual.

[7] Ingalls S., Whitehead A.E., Bridoux K., Tinted M.C. (2010). windows: the presence of the UV absorbing compounds called mycosporine-like amino acids embedded in the frustules of marine diatoms. Geochim. Cosmochim. Acta.

[8] Shieh J., Cho A., Lai Y., Dai B., Pan F., Chao K. (2004). Stable blue luminescence from mesoporous silica films. Electrochem. Solid State Lett..

