# Supplementary figures and images for: Data from two different culture conditions of Thalassiosira weissflogii diatom and from cleaning procedures for obtaining monodisperse nanostructured biosilica
Source: Data Brief. 2016 May 28;8:312–9. doi: 10.1016/j.dib.2016.05.033 (PMC4909712; doi:10.1016/j.dib.2016.05.033)

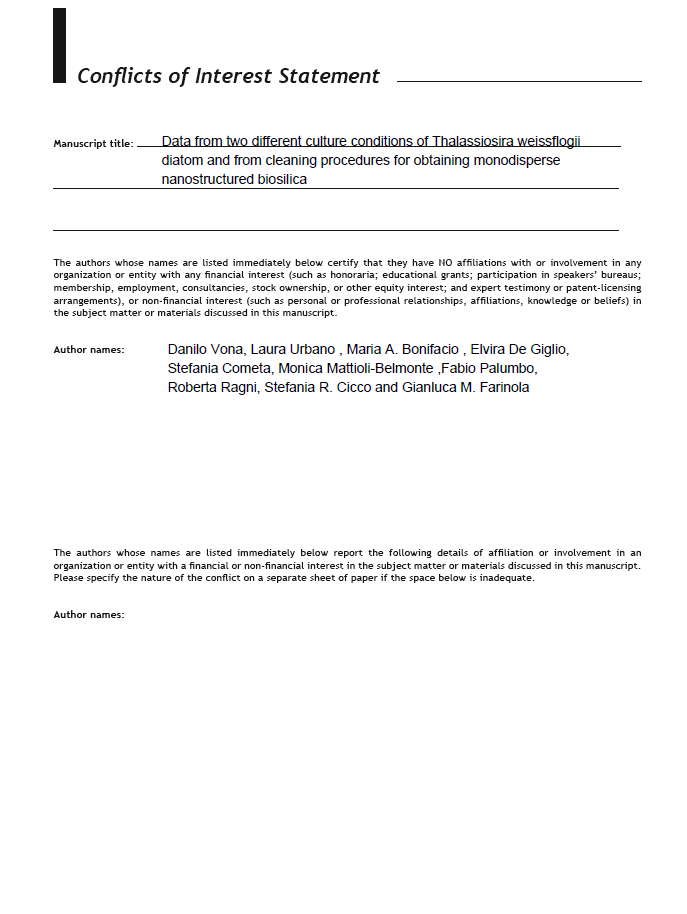

Supplement: Supplementary file 1 — Supplementary material [file mmc1.zip › mmc1/conf_int_1_apr_jpeg.tif]
